# First Branchial Cleft Fistula Associated with External Auditory Canal Stenosis and Middle Ear Cholesteatoma

**Published:** 2014-10

**Authors:** Shahin Abdollahi fakhim, Masoud Naderpoor, Mehrnoosh Mousaviagdas

**Affiliations:** 1 *Department of Otorhinolaryngology, Children Hospital, Tabriz, Iran.*; 2 *Department of Otorhinolaryngology, Imam Reza Hospital, Tabriz, Iran.*

**Keywords:** First branchial cleft, fistula, Canal stenosis, Cholesteatoma.

## Abstract

**Introduction:**

First branchial cleft anomalies manifest with duplication of the external auditory canal.

**Case Report:**

This report features a rare case of microtia and congenital middle ear and canal cholesteatoma with first branchial fistula. External auditory canal stenosis was complicated by middle ear and external canal cholesteatoma, but branchial fistula, opening in the zygomatic root and a sinus in the helical root, may explain this feature. A canal wall down mastoidectomy with canaloplasty and wide meatoplasty was performed. The branchial cleft was excised through parotidectomy and facial nerve dissection.

**Conclusion:**

It should be considered that canal stenosis in such cases can induce cholesteatoma formation in the auditory canal and middle ear.

## Introduction

The branchial apparatus, consisting of arches one through six and their respective clefts and pouches, undergo a complex series of events during embryogenesis (1). First branchial arch abnormalities are rare, accounting for only 1−8% of all branchial anomalies (2). Their aberrant development may lead to the formation of a cervical cyst or sinus in the region of the ear (3). Periauricular cysts or pits occur anterior to the external auditory canal; usually superior to the region of the tragus (3). True branchial cleft anomalies are duplications of the membranous part of the external auditory canal and manifest clinically as cysts, sinuses, or fistulas (3). This error may lead to external auditory canal stenosis and atresia (1). Because of misdiagnosis, management is often inadequate, recurrence is common and iatrogenic injuries of the facial nerve have been reported (4,5). We report an external auditory canal stenosis, and cholesteatoma of the mastoid and middle ear with first branchial fistula to the middle ear from the zygomatic root.

## Case Report

**Fig 1:**
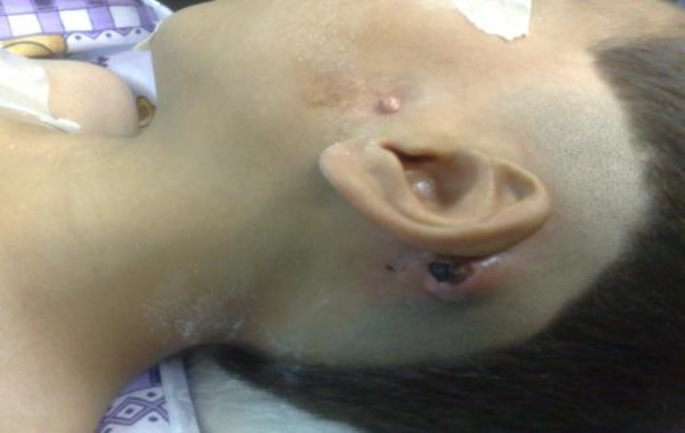
First branchial cleft fistula opening in the middle ear and mastoid cholesteatoma

A 7-year-old boy whose features included microtia, external-ear canal stenosis, congenital cholesteatoma in the middle ear and mastoid with postauricular abscess and automastoidectomy and a first branchial cleft fistula opening in the middle ear through the zygomatic root was referred to our clinic (Fig. 1). 

**Fig. 2:**
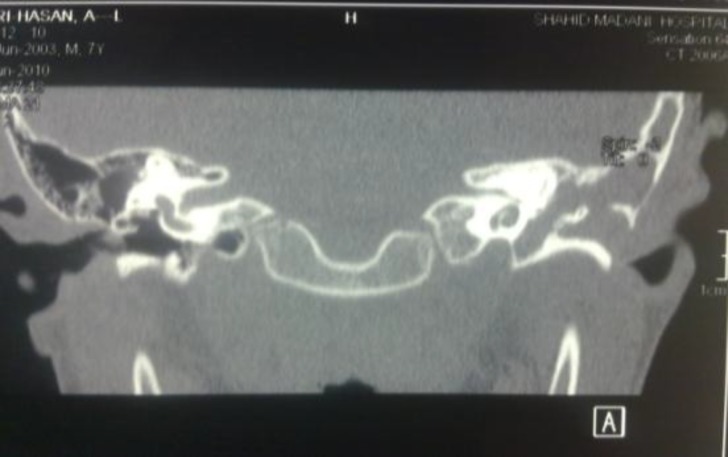
Computed tomography (CT) scan from first branchial cleft fistula opening in the middle ear showing automastoidectomy and the appearance of soft tissue in the middle ear and mastoid

Discharge from the periauricular sinus with wide tract had been occurring since 3−4 years previously. Discharge ended upon antibiotic treatment, but was reported again after cessation of treatment. In a clinical examination, we observed severe auditory canal stenosis and 55-db conductive hearing loss in pure tone audiogram. These results were consistent with tuning tests. Facial nerve function was normal. Imaging studies such as computed tomography (CT) showed the extent of the bony erosion in the mastoid air cells (automastoidectomy) with cortical fistula to the skin. The middle ear and mastoid were filled with the appearance of soft tissue (Fig. 2).

**Fig 3:**
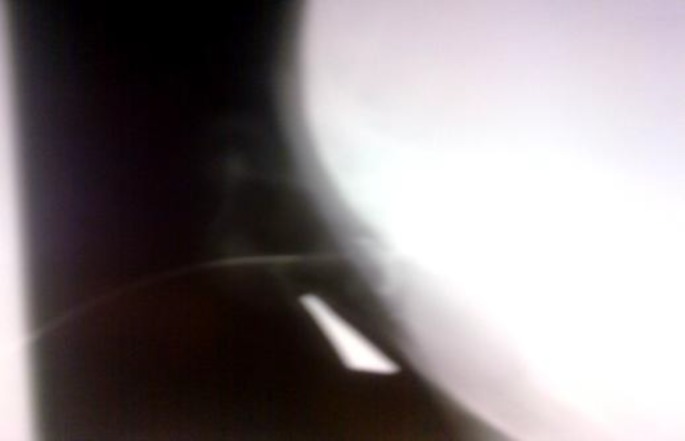
First branchial fistula and its tract to the zygomatic root shown via fistulography

The first branchial fistula and its tract to the zygomatic root were shown by fistulography (Fig.3). 

**Fig. 4:**
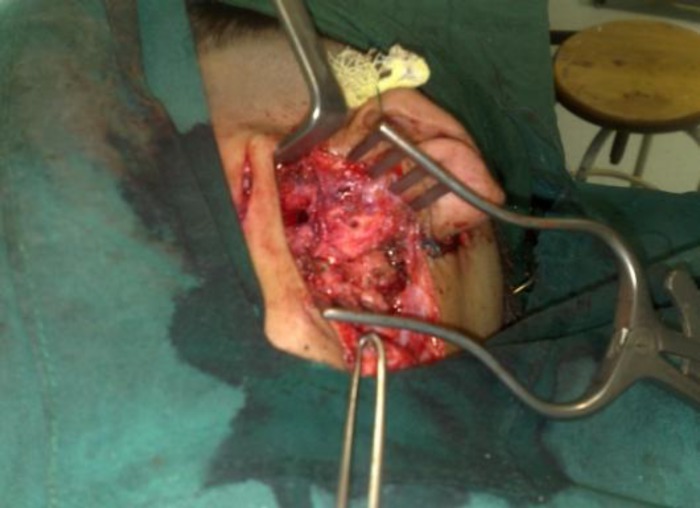
Intraoperative view of parotidectomy and fistula resection in the case of branchial fistula

An exploration of the mastoid through a postauricular approach, with the fistula site in the incision, was planned. One large mastoid cavity was found to be full of cholesteatoma, extending from the anterior wall of the middle ear where the branchial fistula was opening and completely obliterating the external auditory canal, middle ear and mastoid cells with ossicular chain erosion. Canal wall down mastoidectomy with canaloplasty and wide meatoplasty was performed. The Eustachian tube was also cleaned completely. The branchial cleft fistula from the anterior auricle to the zygomatic root was excised through parotidectomy and facial nerve dissection (Fig. 4). 

The tract of the first branchial fistula crossed the superior branch of the facial nerve. There was a hole in the zygomatic root in which the epithelium was extended to the middle ear. The tract was excised completely and confirmed pathologically as squamous epithelium. The site of the operation was closed. Facial nerve function was normal in the post-operative period. No evidence of recurrence was observed during a 6-month follow-up period.

## Discussion

At 5 weeks of gestation, the area of the developing face and neck of the embryo consists of five or six pairs of finger-like masses of tissue named the branchial arches (3). The first arch forms the maxilla, mandible, incus, malleus, muscles of mastication, the anterior two-thirds of the tongue and part of the pina. The first cleft gives rise to the external auditory canal and the lateral surface of the tympanic membrane (6). 

First branchial cleft anomalies are categorized into type 1 and 2 lesions based on clinical features and histological findings. However, not all lesions fit into this classification system. Furthermore, this classification system does not address cases of external auditory canal stenosis and atresia, which are also disorders of first branchial cleft development (7). Potential developmental defect is failure of obliteration of the ventral aspect of the first cleft during weeks 5 and 6. This results in a duplication anomaly of the external auditory canal (7). The most common presentation of a patient with a type-1 first branchial cleft anomaly is recurrent periauricular swelling or abscess formation, often treated with incision and drainage alone (8). Type 2 anomalies commonly produce symptoms in the first year of life, with sinus tract presenting as otorrhea or periauricular drainage (9). 

In our case, there was left external auditory canal stenosis, periauricular sinus and microtia with cholesteatoma in all mastoid air cells and the middle ear. Cholesteatoma was derived from the sinus tract to the middle ear and completely filled the mastoid and Eustachian tube, forming an ossicular and bony erosion, and postauricular abscess. Yalcin et al. (10) reported a case of first branchial sinus presenting with cholesteatoma and external auditory canal atresia. Lee et al. (11) reported the first branchial anomalies and cholesteatoma which makes a bony defect in the mastoid segment of the facial canal. Surgeons should have a suspicion of cholesteatoma destruction in this case. If cholesteatoma is present, surgery should not be delayed. In our case, cholesteatoma was removed completely with canal wall down mastoidectomy and wide meatoplasty and reconstruction of stenotic canal, then the sinus tract was incised completely with facial dissection by superficial parotidectomy. External auditory canal stenosis was repaired at the time of surgery with no evidence of cholesteatoma recurrence.

## Conclusion

First branchial sinus anomaly can present with external auditory canal stenosis and cholesteatoma. In this case, the middle ear and mastoid must be examined to rule out this manifestation. 
